# Bleb Compressive Sutures in the Management of Hypotony Maculopathy after Glaucoma Surgery

**DOI:** 10.3390/jcm10112223

**Published:** 2021-05-21

**Authors:** Ewa Kosior-Jarecka, Dominika Wróbel-Dudzińska, Anna Święch, Tomasz Żarnowski

**Affiliations:** 1 Department of Diagnostics and Microsurgery of Glaucoma, Medical University of Lublin, 20-059 Lublin, Poland; dwrobeldudzinska@interia.pl (D.W.-D.); zarnowskit@poczta.onet.pl (T.Ż.); 2 Department of Vitreoretinal Surgery, Medical University of Lublin, 20-059 Lublin, Poland; anna.zub@umlub.pl

**Keywords:** hypotony maculopathy, complications of antiglaucoma surgery, bleb compressive sutures

## Abstract

PURPOSE: The aim of the study was to assess the efficacy and safety of compressive sutures in patients with hypotony maculopathy after glaucoma surgery. METHODS: This retrospective case series analyzes the clinical outcomes of conjunctival compressive sutures in 17 patients with hypotony maculopathy developed after glaucoma surgery. Compressive Nylon 10–0 single sutures were used in all patients; in two patients, the procedure was repeated. All patients underwent ophthalmic evaluation and macular OCT scanning before the surgery, one month, six months, and one year after the procedure. RESULTS: Mean intraocular pressure (IOP) before suturing was 2.3 ± 1.57 mmHg and increased to 14.2 ± 7.03 mmHg (*p* = 0.00065) one month after the procedure. After six months, mean IOP was 10.2 ± 4.3 mmHg (*p* = 0.005), and after one year ± 4.7 mmHg (*p* = 0.0117). To obtain the target pressure, the sutures had to be removed in one patient, and medical therapy was undertaken in three patients. Mean decimal best-corrected visual acuity (BCVA) before the sutures was 0.18 ± 0.13 and increased to 0.53 ± 0.25 (*p* = 0.0004) after one month, to 0.46 ± 0.31 (*p* = 0.005) after six months, and to 0.31 ± 0.22 (*p* = 0.025) after one year. In one case, leakage from the bleb was observed after the procedure and bleb revision was required. CONCLUSIONS: transconjuctival compressive sutures seem to be an efficient and safe technique for managing hypotony maculopathy after glaucoma surgery.

## 1. Introduction

Glaucoma, one of the leading causes of irreversible blindness worldwide [1], is a progressive optic neuropathy, and elevated intraocular pressure (IOP) is one of the main risk factors for the development and progression of this disease [2]. A decrease of IOP remains the only clinical method with confirmed efficacy for diminishing the progression rate of glaucoma, and this effect can be achieved by means of medical treatment, laser, or surgery [3].

After trabeculectomy, a standard surgical glaucoma procedure, aqueous humor flows into the surgically created filtering bleb [4]. The final success of trabeculectomy depends not only on the surgical technique but also on the possibility of slowing down the healing processes [5]. Therefore, the challenge faced by both surgical technique and postsurgical care is to find a balance between IOP low enough to obtain the target pressure and at the same time high enough to avoid complications related to ocular hypotony [6]. Additionally, not every case of post-surgical low IOP level leads to ocular complications [7].

Clinical hypotony is defined as a level of IOP that is too low to maintain the shape of the eyeball and results in structural and functional changes [8,9]. If left untreated, prolonged hypotony may cause various serious complications such as bleb infection, cataract formation, synechiae, persistent choroidal detachment, or hypotony maculopathy [10]. Hypotony maculopathy and bleb infection in the course of clinical hypotony are potentially sight-threatening [11,12].

The clinical characteristics of hypotony maculopathy were first described by Dellaporta in 1954 as “creasing of retina in hypotonia” [13], but the modern definition of hypotony maculopathy was introduced by Gass to emphasize the etiology of vision loss in the setting of chorioretinal folds [14]. Macular hypotony is characterized by a decrease in visual acuity caused by macular folds, retinal edema, papilledema, and vascular tortuosity. Structurally, it is believed that low IOP level causes thickening of the perifoveal choroid and sclera, which results in their central displacement, visible as macular folds. Over time, these changes cause photoreceptor damage and become irreversible, which can limit recovery of visual function even after restoration of normal IOP [15,16].

Treatment options for hypotony after glaucoma surgery mainly caused by overfiltrating blebs include conservative management of topical autologous serum [17], bleb injection of autologous blood [18] or viscoelastic material [19], and anterior chamber injection of viscoelastic material [20] or gas [21]. Conservative management usually has minor and only short-lasting effects. Surgical management includes transconjunctival flap suturing [22,23], excision of thin blebs and conjunctival advancement [16], patch grafting using donor sclera [24,25], donor cornea [26], and autologous conjunctiva [27,28]. The variety of described techniques shows that all these procedures have their disadvantages. On the other hand, the techniques that reduce the transscleral flow by suturing or tissue patching may lead to limitation of the outflow resulting in very high IOP elevation [29].

Transconjunctival compressive sutures were introduced to the management of post-surgical hypotony as a simple and effective technique [30]. The aim of the study was to evaluate the efficacy and safety of transconjunctival suturing of overfiltrating blebs in hypotony maculopathy after glaucoma surgery.

## 2. Materials and Methods

The studied group consisted of 17 Caucasian patients with hypotony maculopathy after glaucoma surgery treated in the Department of Diagnostics and Microsurgery of Glaucoma, Medical University of Lublin, Poland, between 2015 and 2017. During this period, every patient who met inclusion criteria participated in the study.

The inclusion criteria was as follows:

- Age at glaucoma diagnosis of over 18 years;
- At least 6 months after the antiglaucoma procedure;
- Clinically significant hypotony: intraocular pressure (IOP) lower than 6 mmHg, associated with the BCVA decreased by at least 2 lines on Snellen charts in comparison to pre-trabeculectomy results;
- Features of hypotony maculopathy with macular folds;
- No progression of the cataract;
- No leakage from the bleb;
- No kissing choroidal effusion.

Clinical and demographic characteristics of the studied group are presented in Table 1. The studied group included 7 patients with primary open angle glaucoma (POAG), 4 with glaucoma in the course of pigment dispersion syndrome (PDSG), 4 with pseudo-exfoliative glaucoma (PEXG) and 1 with primary angle closure glaucoma (PACG).

The information about patients’ medical history was obtained from their clinical records. At the inclusion visit, patients underwent ophthalmic examination with BCVA (decimal Snellen charts), Goldman applanation tonometry, slit lamp examination, eye fundus assessment by ophthalmoscopy, and OCT (Stratus, Carl Zeiss Meditec, Dublin, Ireland) measurements assessing the thickness of the macula and the presence of choroidal folds.

**Scheme 1:**
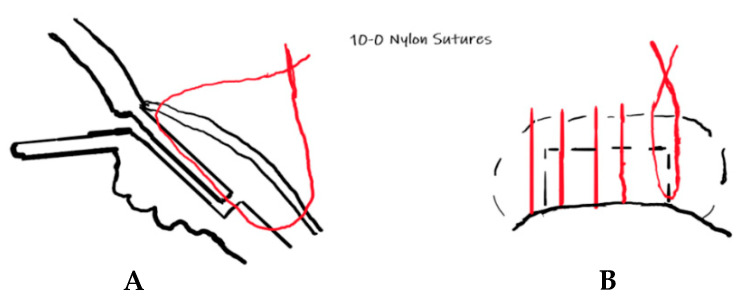
Transconjunctival sutures placement: **A**, side view; **B**, front view.

During the surgical procedure, 5–7 single Nylon 10–0 sutures were placed transconjunctivally in the area of the bleb as described earlier [30]. In brief, after peribulbar anesthesia, the bulb was rotated downward and single sutures were placed, starting from the limbus and extending posteriorly as far as possible toward the superior fornix. During suturing, attempts were made to catch not only the conjunctiva but also a part of the underlying sclera, if the height of the bleb allowed for it. The sutures were intended to be placed on the area of existing scleral flap or in its proximity. During the procedure, after placing the 5th suture, paracentesis was performed and the BSS was administered into the anterior chamber to assess the increase of IOP and the reduction of the outflow. Further sutures were added until the increase in IOP was observed (up to 7 sutures). The idea and the surgical technique are shown in Scheme 1.

In the post-surgical period, only fixed combination of dexamethasone/tobramycine was used in decreasing doses (starting from 4 times a day, and decreasing 1 drop per week up to 4 weeks when the drops were stopped). Some patients needed to have installed the preservatives free lubricant drops.

The patients were controlled one day after the procedure, as well as 7 days, 1 month, 3 months, 6 months, and 12 months after the procedure. During the check-ups, ophthalmic examination was performed including BCVA testing (decimal Snellen charts), Goldman applanation tonometry, slit lamp examination, eye fundus assessment by ophthalmoscopy, and OCT measurements assessing the thickness of the macula and the presence of choroidal folds. All patients were present at all time-point visits. However, in 2 cases at 6 months, OCT was not performed because of technical problems. Additionally, the study eye was examined at every visit to look for possible complications.

Two criteria of success were defined:

1. IOP over 6 mmHg;
2. BCVA improvement of at least 2 lines on a Snellen chart.

Changes in the assessed values (BCVA and IOP) were measured at each control visit planned in the study by subtracting the preoperative value from the postoperative value. Statistic evaluation of the data was performed using Statistica 13.1 (Polish version, Statsoft Poland). The results were reported mainly as mean ± SD or percentage values. A *p*-value lower than 0.05 was considered statistically significant. Normal distribution was assessed with the Shapiro–Wilk test. The Mann–Whitney test was used for non-normally distributed data.

## 3. Results

**Figure 1:**
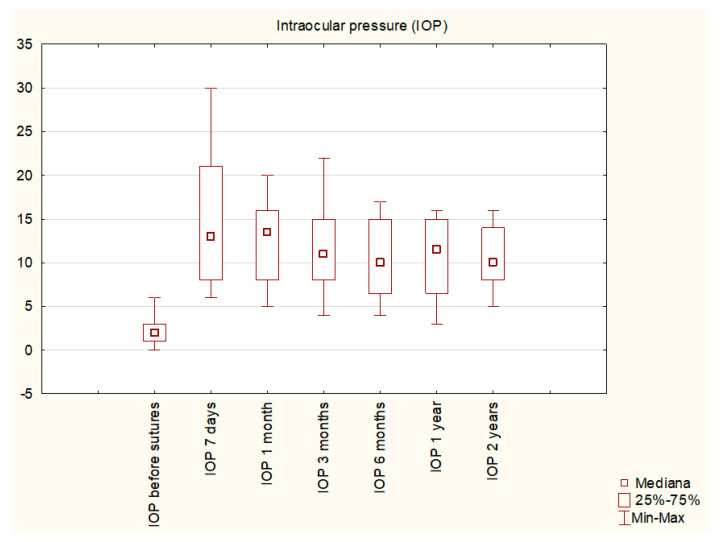
Changes in the intraocular pressure during the study period.

Mean IOP before suturing was 2.3 ± 1.57 mmHg and increased to 14.2 ± 7.03 mmHg (*p* = 0.00065) at 1 month post-op. After 6 months, mean IOP was 10.2 ± 4.3 mmHg (*p* = 0.005); and after one year ± 4.7 mmHg (*p* = 0.0117) (Figure 1). On day 7 after surgery, IOP value exceeded 21 mmHg in 4 (23.5%) patients, with a value of over 30 mmHg in one case. To obtain the target pressure, one patient needed to have one suture removed, and medical therapy was undertaken in three patients.

Success criterion 1 (IOP over 6 mmHg) at 7 days post-procedure was achieved in 15 (88%) patients, after 3 months in 10 (59%) patients, and after 6 months in 9 (53%) patients (and remained stable during the first and second year of follow-up).

**Figure 2:**
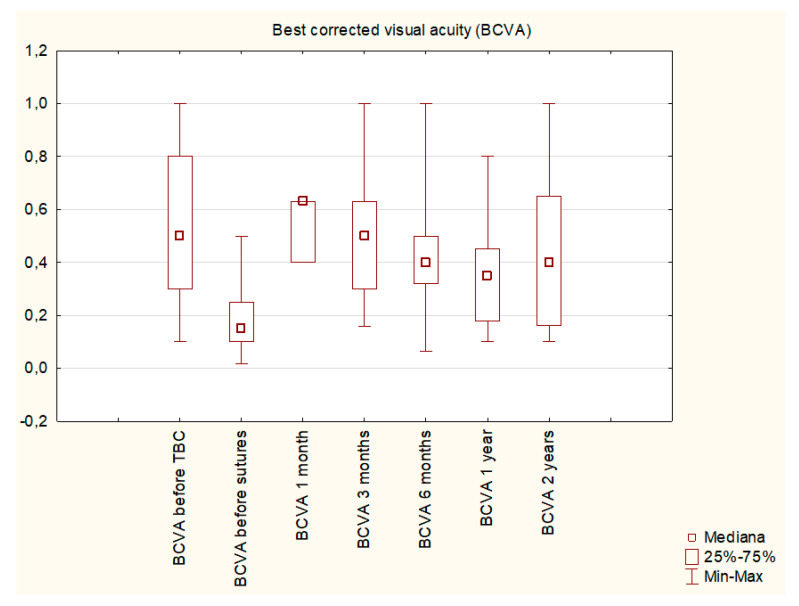
Changes in best-corrected visual acuity during the study period.

Mean BCVA before applying the sutures was 0.18 ± 0.13 and increased to 0.53 ± 0.25 (*p* = 0.0004) after 1 month; to 0.46 ± 0.31 (*p* = 0.005) after 6 months; and to 0.31 ± 0.22 (*p* = 0.025) after one year (Figure 2).

Success Criterion 2 (BCVA improvement of at least 2 lines) was fulfilled in the whole studied group at 3 months post-procedure, in 14 patients (82%) at 6 months, in 12 (71%) at 1 year, and in 11 (65%) at 2 years.

**Figure 3:**
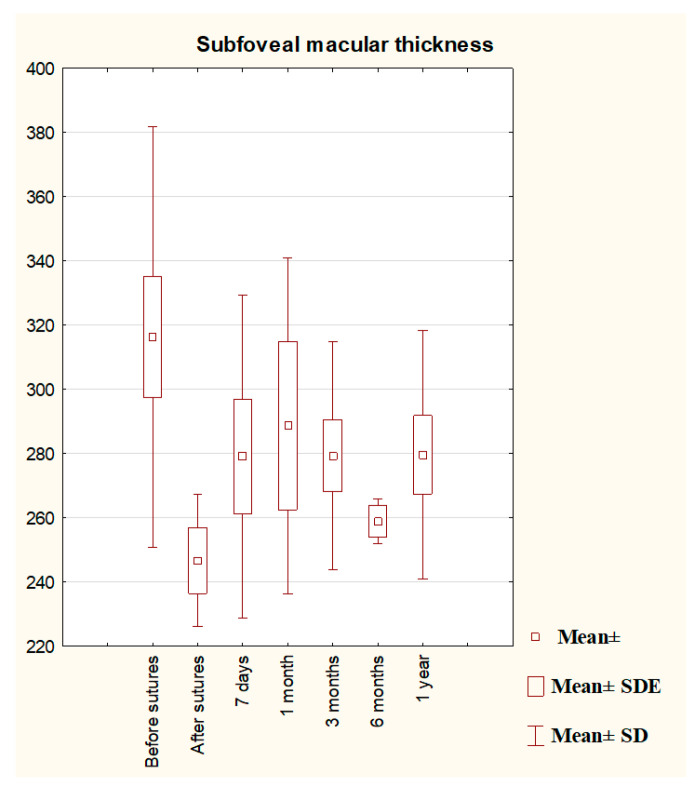
Changes in the subfoveal macular thickness assessed by optical coherent tomography during the study period.

Mean subfoveal macular thickness before suturing was 316.33 μm, and decreased to 283.22 μm at 1 month (*p* = 0.0314), and to 279.77 μm at 1 year (*p* = 0.0322) (Figure 3). Macular folds were present in every patients at the inclusion. Starting from 3rd month macular folds were not observed during ophthalmoscopy in 7 patients (41.1%).

No significant correlations were found between the change in IOP and the change in mean subfoveal macular thickness in OCT, except for the tendency to a negative correlation between these results obtained at 6 months (*p* = 0.1050; R = −0.61). Interestingly, we could not find any significant correlations between the change in macular thickness in OCT and the change in BCVA (*p* = 0.9329 at 3 months; *p* = 0.2682 at 6 months, and 0.644512 at 1 year).

The mean time between the primary surgery and compression suturing was 3.08 years. The time between the primary surgery and suturing did not correlate with postoperative IOP (*p* = 0.9537 at 1 month; *p* = 0.5181 at 3 months, *p* = 0.2333 at 6 months, and *p* = 0.6895 at 1 year). Assessing the influence of the time since the primary procedure on the final BCVA, we observed negative tendencies at 3 and 6 months (*p* = 0.2725, R = −0.34, and *p* = 0.1553, R = −0.51, respectively) and a significant negative correlation at 1 year (*p* = 0.0138; R = −0.94).

When our group was divided into 2 subgroups: with time from the primary surgery shorter than one year (early group: 8 patients) and longer than a year (late group: 9 patients), no differences in BCVA were found (late: 0.18 vs. early: 0.16, *p* = 0.2470 before sutures; late: 0.33 vs. early: 0.37, *p* = 0.7881 at 1 year). The initial IOP did not differ the groups (late: 1.67 mmHg vs. early: 2.65 mmHg; *p* = 0.3799). However, the IOP seemed to be higher in early group with statistical tendency at 1 year (late: 7.33 mmHg vs. early: 11.78; *p* = 0.1671) and statistical significance at 2 years (late: 6.66 mmHg vs. early: 12.5 mmHg; *p* = 0.0067).

Two patients had peripheral choroidal detachments at the inclusion, which resolved within 7 days post-op. In one case, leakage from the bleb was observed after the procedure, and bleb revision procedure was needed.

**Figure 4:**
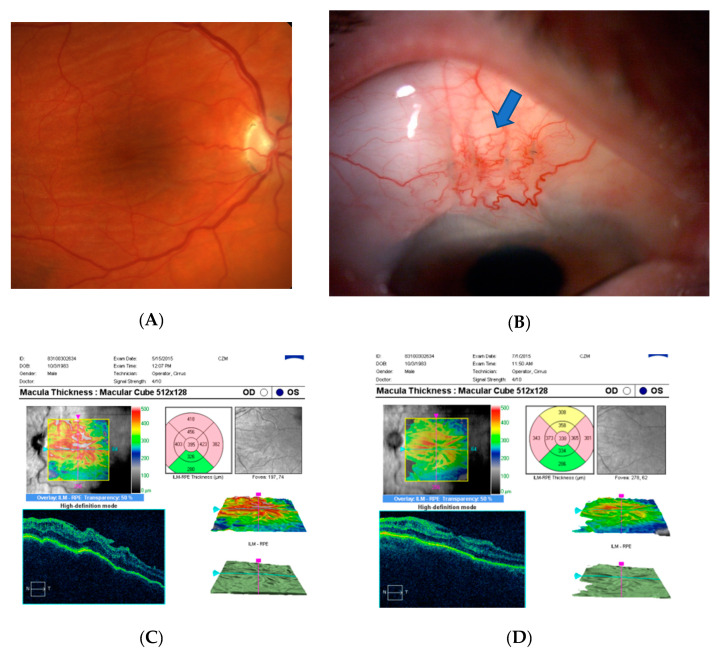
Sample case. (**A**) Eye fundus with hypotony maculopathy; (**B**) compressive sutures—1 day after the surgery (blue arrow points the sutures); (**C**) macular OCT before suturing; (**D**) macular OCT after suturing.

The exemplary case is presented on Figure 4.

## 4. Discussion

The wide range of reported chronic hypotony after antiglaucoma procedures is due to the lack of a standardized definition [31,32]. Additionally, some eyes require very low IOP to stop or slow down glaucoma progression [33]. In cases when pre-surgery IOP has lower values or when glaucomatous optic neuropathy is advanced, an IOP below 6 mm Hg without cataract progression, choroidal effusion, or maculopathy, and with improved glaucoma is considered a surgical success [34]. In this study, transconjunctival suturing was performed when the IOP maintained under 6 mmHg and the BCVA remained significantly decreased due to hypotony maculopathy.

The perfect time for intervention in case of hypotony after antiglaucoma procedure is also not clear. The tendency to delay the treatment is observed because clinical experience suggests that most eyes with chronic hypotony following a trabeculectomy maintain good visual acuity without complications [11]. Moreover, spontaneous recovery from hypotony caused by natural healing processes is frequent [34]. Additionally, a lot of procedures designed to cure hypotony turn out to be unsuccessful: 5 of our patients had previously had blood injected to the bleb to enhance healing without prolonged effect.

The duration of hypotony has been reported to have no correlation with final visual outcomes [11,35]. In the case of our patients with hypotony maculopathy, we observed that the time since the primary procedure indeed did not influence the final IOP level after transconjunctival suturing. If timely managed, IOP increase usually leads to the restoration of the normal smooth architecture of the retina, allowing for realignment of photoreceptors and visual recovery [31]. On the other hand, prolonged hypotony causes irreversible fibrosis within the retina, choroid, or sclera, maintaining the choroid in a folded position [31]. Scoralick et al. found resolution of hypotony maculopathy in 85% of the cases following surgical reintervention, but none of the variables investigated (including the time interval between trabeculectomy and flap resuturing) was significantly associated with postoperative maculopathy resolution [36].

Numerous risk factors for hypotony after glaucoma surgery have been identified, including myopia, young age, antimetabolite use, pre-existing inflammation, aphakia, and old age accompanied by a thin conjunctiva and thinner CCT [11,37,38]. Besides the application of an antifibrotic agent, male gender, high myopia, young age, and patients receiving primary filtering surgery have also been associated with an increased risk of hypotony maculopathy [39,40]. In our study, five (29%) patients underwent primary procedure at the age below 40 because of pigmentary glaucoma or traumatic glaucoma. It confirms the susceptibility of younger patients to hypotony maculopathy. Additionally, all of these patients were myopic males, which suggests that care needs to be taken in this group during the primary procedure to avoid hypotony, with lower titers of antimetabolites and tight bleb suturing. Lower scleral rigidity in younger patients is believed to be related to the development of hypotony maculopathy [14,40]. Additionally, myopic eyes tend to have thinner sclera, which is related to a general loss of collagen and proteoglycans [41] and makes sclera more vulnerable to collapse during hypotony. Further, males tend to have lower scleral rigidity and a correlation between male gender and hypotony maculopathy has been ascertained [42].

In our practice, transconjunctival sutures are an effective and safe method of increasing IOP and improving visual acuity in patients with hypotony maculopathy as a complication of antiglaucoma procedure. In a retrospective study, Letarte et al. showed a significant IOP increase and BCVA improvement six months after transconjunctival scleral flap resuturing [43]. Eha et al. [22,44] published a prospective case study describing the outcome of 16 patients whose mean IOP was 9.6 mmHg and mean BCVA was 20/60, six months following the procedure. These results are similar to ours with mean IOP value of 9 mmHg 1 year after suturing. We observed a gradual decrease in mean IOP in the course of the study, with the highest values one month postoperatively.

Although the success in IOP increase was not observed in every patient, during the early postoperative period we observed an improvement of the visual acuity in the whole group, similarly to the results observed by Scoralick [36]. It shows the efficacy of transconjunctival sutures regarding BCVA improvement, and probably additionally confirms that numerous definitions of hypotony are not accurate, as in some patients an increase in IOP even below 6 mmHg allowed for BCVA improvement.

In our group, but not in every case, it was possible to obtain the stable IOP increase in post-surgical period, which may have some possible reasons. First, it may be connected with not enough traction obtained during placement of the sutures, which is usually connected with the changes of the ocular tissues during early postsurgical period (for example, the decrease in conjunctival oedema or moving of the sutures through the conjunctiva). Tight suturing with the attachment to the superficial parts of the sclera in case of extensively hyperfiltarating and elevated blebs may also be problematic. Additionally, it is possible that after MMC application during primary procedure the scleral tissues are weakened and melted which makes transconjunctival suturing not enough for limitation of the outflow. In such cases, the revision with opening of conjunctiva and direct suturing of the sclera may be beneficial. Finally, after antiglaucoma procedure prolonged ciliary body hypoperfusion and hyposecretion is possible.

Clinical assessment had led us to the assumption that a shorter time since the primary procedure may enable obtaining a more prominent increase in the IOP level, which was not confirmed by our results: in this study, at no time-point was IOP correlated with the time since the primary surgery, which was also observed by other authors [36]. However, in our results a longer time since the primary surgery tended to have a negative impact on the final BCVA. It may be related to the observation that when left untreated, hypotony maculopathy can have long-term visual consequences [7] with irreversible chorioretinal folds resulting from fibrosis within the retina, choroids, or sclera [31,45]. Additionally, in our study the patients with sutures placed earlier tended to have higher IOP values in longer observation period. This is why our results may provide an argument for an earlier intervention in maculopathy hypotony. However, in our opinion, the surprising finding of this study is the lack of correlation between the improvement in BCVA and the changes in subfoveal macular thickness determined in OCT. It may be explained by the fact that during the study, intra- or sub-retinal fluid was not observed; thus, the initial BCVA decrease after the antiglaucoma procedure was not caused by any disturbances in the morphology of the retinal layers but rather by their folding. Additionally, the improvement in BCVA may be partially explained by the restoration of the anterior segment structures after the increase in IOP [46,47,48]. The changes in the hemodynamics of the choroid affected by the IOP increase may also be involved in BCVA improvement [49].

In general, the highest IOP during the follow-up period was measured on postoperative Day 7. Furthermore, this spike in IOP seemed to be beneficial for expediting the resolution of any preexisting serous choroidal detachments [16] and cause quicker improvement in BCVA, as observed in this study. However, in such cases, the improvement of the visual acuity was not stable; all three patients with the decrease in BCVA observed at six months belonged to this group. The highest values of early postoperative IOP were observed in the group of younger patients with pigmentary glaucoma, which may also be connected with scleral vulnerability, as mentioned earlier. On the other hand, such high spikes in IOP may be potentially harmful in the case of severely damaged visual field in advanced glaucoma.

Except for the observed IOP spikes, transconjunctival sutures seem to be a safe procedure. In the course of the study, we observed only one patient with avascular thin bleb who suffered from bleb leakage and needed bleb revision, which involved covering it with the mobilized conjunctiva.

To sum up, transconjunctival sutures seem to be an effective and safe method to treat hypotony maculopathy after glaucoma surgery. In the study, the improvement in BCVA started with the elevation of IOP at the early postoperative period and remained stable during the observation time. Our results also provide an argument for an earlier intervention in case of clinical hypotony after glaucoma surgery.

## Figures and Tables

**Table 1:** Clinical and demographic characteristics of the studied group.

Feature	n/Mean Value ± SD
Number of patients	17
Gender	8F/10 M
Age	60.5 ± 20.5
Diagnosis	POAG: 7 cases; PDSG: 4 cases: PEXG: 4 cases; PACG: 1 case; traumatic glaucoma: 1 case.
Primary procedure	Trabeculectomy: 9 cases; Phacotrabeculectomy: 4 cases; Needle revision: 3 cases; Deep sclerectomy: 1 case.
Mean IOP before primary procedure	33.6 ± 11.6 mmHg
Mean IOP before sutures	2.3 ± 1.57 mmHg
BCVA before primary procedure	0.55 ± 0.31
BCVA before sutures	0.18 ± 0.13
Mean MD	16.21 ± 7.45 dB
Mean time between primary procedure and compression sutures	3.08 years

POAG, primary open angle glaucoma; PDSG, pigment dispersion syndrome; PEXG, pseudo-exfoliative glaucoma; PACG, primary angle closure glaucoma; IOP, mean intraocular pressure; BCVA, best-corrected visual acuity; MD, mean deviation.

## Data Availability

Data are available from the corresponding author on demand.

## References

[1] Quigley H.A., Broman A.T. (2006). The number of people with glaucoma worldwide in 2010 and 2020. Br. J. Ophthalmol..

[2] Weinreb R.N., Aung T., Medeiros F.A. (2014). The pathophysiology and treatment of glaucoma: A review. JAMA.

[3] Weinreb R.N., Khaw P.T. (2004). Primary open-angle glaucoma. Lancet.

[4] Khaw P.T., Chiang M., Shah P., Sii F., Lockwood A., Khalili A. (2017). Enhanced Trabeculectomy: The Moorfields Safer Surgery System. Dev. Ophthalmol..

[5] Joseph J.P., Miller M.H., Hitchings R.A. (1988). Wound healing as a barrier to successful filtration surgery. Eye.

[6] Costa V.P., Arcieri E.S. (2007). Hypotony maculopathy. Acta Ophthalmol. Scand..

[7] Seah S.K., Prata J.A., Minckler D.S., Baerveldt G., Lee P.P., Heuer D.K. (1995). Hypotony following trabeculectomy. J. Glaucoma.

[8] Pederson J.E., Ritch R., Krupin T., Shields M.B. (1996). Ocular Hypotony. The Glaucomas.

[9] Rahman A., Mendonca M., Simmons R.B., Simmons R.J. (2000). Hypotony after glaucoma filtration surgery. Int. Ophthalmol. Clin..

[10] Edmunds B., Thompson J.R., Salmon J.F., Wormald R.P. (2002). The National Survey of Trabeculectomy. III. Early and late complications. Eye.

[11] Saeedi O.J., Jefferys J.L., Solus J.F., Jampel H.D., Quigley H.A. (2014). Risk factors for adverse consequences of low intraocular pressure after trabeculectomy. J. Glaucoma.

[12] Francis B.A., Hong B., Winarko J., Kawji S., Dustin L., Chopra V. (2011). Vision loss and recovery after trabeculectomy: Risk and associated risk factors. Arch. Ophthalmol..

[13] Dellaporta A. (1954). Creasing of retina in hypotonia. Klin. Mon. Augenheilkd. Augenarztl. Fortbild..

[14] Gass J.D., Bellows J.G. (1972). Hypotony Maculopathy. Contemporary Ophthalmology: Honoring Sir Stewart Duke-Elder.

[15] Oyakhire J.O., Moroi S.E. (2004). Clinical and anatomical reversal of long-term hypotony maculopathy. Am. J. Ophthalmol..

[16] Bitrian E., Song B.J., Caprioli J. (2014). Bleb revision for resolution of hypotony maculopathy following primary trabeculectomy. Am. J. Ophthalmol..

[17] Matsuo H., Tomidokoro A., Tomita G., Araie M. (2005). Topical application of autologous serum for the treatment of lateonset aqueous oozing or point-leak through filtering bleb. Eye.

[18] Smith M.F., Magauran R.G., Betchkal J., Doyle J.W. (1995). Treatment of postfiltration bleb leaks with autologous blood. Ophthalmology.

[19] Higashide T., Tagawa S., Sugiyama K. (2005). Intraoperative Healon5 injection into blebs for small conjunctival breaks created during trabeculectomy. J. Cataract Refract. Surg..

[20] Hosoda S., Yuki K., Ono T., Tsubota K. (2013). Ophthalmic viscoelastic device injection for the treatment of flat anterior chamber after trabeculectomy: A case series study. Clin. Ophthalmol..

[21] Kurtz S., Leibovitch I. (2002). Combined perfluoropropane gas and viscoelastic material injection for anterior chamber reformation following trabeculectomy. Br. J. Ophthalmol..

[22] Eha J., Hoffmann E.M., Wahl J., Pfeiffer N. (2008). Flap suture—A simple technique for the revision of hypotony maculopathy following trabeculectomy with mitomycin C. Graefes Arch. Clin. Exp. Ophthalmol..

[23] Shirato S., Maruyama K., Haneda M. (2004). Resuturing the sclera flap through conjunctiva for treatment of excess filtration. Am. J. Ophthalmol..

[24] Haynes W.L., Alward W.L. (1995). Rapid visual recovery and longterm intraocular pressure control after donor scleral patch grafting for trabeculectomy-induced hypotony maculopathy. J. Glaucoma.

[25] Harizman N., Ben-Cnaan R., Goldenfeld M., Levkovitch-Verbin H., Melamed S. (2005). Donor scleral patch for treating hypotony due to leaking and/or overfiltering blebs. J. Glaucoma.

[26] Bochmann F., Kaufmann C., Kipfer A., Thiel M.A. (2014). Corneal patch graft for the repair of late-onset hypotony or filtering bleb leak after trabeculectomy: A new surgical technique. J. Glaucoma.

[27] Panday M., Shantha B., George R., Boda S., Vijaya L. (2011). Outcomes of bleb excision with free autologous conjunctival patch grafting for bleb leak and hypotony after glaucoma filtering surgery. J. Glaucoma.

[28] Dietlein T.S., Lappas A., Rosentreter A. (2013). Secondary subconjunctival implantation of a biodegradable collagen-glycosaminoglycan matrix to treat ocular hypotony following trabeculectomy with mitomycin C. Br. J. Ophthalmol..

[29] Laspas P., Wahl J., Peters H., Prokosch-Willing V., Chronopoulos P., Grehn F., Pfeiffer N., Hoffmann E.M. (2021). Outcome of Bleb Revision With Autologous Conjunctival Graft Alone or Combined With Donor Scleral Graft for Late-onset Bleb Leakage with Hypotony after Standard Trabeculectomy with Mitomycin C. J. Glaucoma.

[30] Maruyama K., Shirato S. (2008). Efficacy and safety of transconjunctival scleral flap resuturing for hypotony after glaucoma filtering surgery. Graefes Arch. Clin. Exp. Ophthalmol..

[31] Jampel H.D., Pasquale L.R., Dibernardo C. (1992). Hypotony maculopathy following trabeculectomy with mitomycin C. Arch. Ophthalmol..

[32] Thomas M., Vajaranant T.S., Aref A.A. (2015). Hypotony maculopathy: Clinical presentation and therapeutic methods. Ophthalmol. Ther..

[33] Higashide T., Ohkubo S., Sugimoto Y., Kiuchi Y., Sugiyama K. (2016). Persistent hypotony after trabeculectomy: Incidence and associated factors in the Collaborative Bleb-Related Infection Incidence and Treatment Study. Jpn. J. Ophthalmol..

[34] Yun S., Chua B., Clement C.I. (2015). Does Chronic Hypotony following Trabeculectomy Represent Treatment Failure?. J. Curr. Glaucoma Pract..

[35] Cohen S.M., Flynn H.W., Palmberg P.F., Gass J.D., Grajewski A.L., Parrish R.K. (1995). Treatment of hypotony maculopathy after trabeculectomy. Ophthalmic Surg. Lasers.

[36] Scoralick A.L.B., Almeida I., Ushida M., Dias D.T., Dorairaj S., Prata T.S., Kanadani F.N. (2017). Hypotony Management through Transconjunctival Scleral Flap Resuturing: Analysis of Surgical Outcomes and Success Predictors. J. Curr. Glaucoma Pract..

[37] Stamper R. (2001). Bilateral chronic hypotony following trabeculectomy with mitomycin C. J. Glaucoma.

[38] Silva R.A., Doshi A., Law S.K., Singh K. (2010). Postfiltration hypotony maculopathy in young chinese myopic women with glaucomatous appearing optic neuropathy. J. Glaucoma.

[39] Singh K., Byrd S., Egbert P.R., Budenz D. (1998). Risk of hypotony after primary trabeculectomy with antifibrotic agents in a black West African population. J. Glaucoma.

[40] Fannin L.A., Schiffman J.C., Budenz D.L. (2003). Risk factors for hypotony maculopathy. Ophthalmology.

[41] Curtin B.J., Iwamoto T., Renaldo D.P. (1979). Normal and staphylomatous sclera of high myopia. An electron microscopic study. Arch. Ophthalmol..

[42] Nicolela M.T., Carrillo M.M., Yan D.B., Rafuse P.E. (2007). Relationship between central corneal thickness and hypotony maculo-pathy after trabeculectomy. Ophthalmology.

[43] Letartre L., Basheikh A., Anctil J.-L., Des Marchais B., Goyette A., Kasner O.P., Lajoie C. (2009). Transconjunctival suturing of the scleral flap for overfiltration with hypotony maculopathy after trabeculectomy. Can. J. Ophthalmol..

[44] Eha J., Hoffmann E.M., Pfeiffer N. (2013). Long-term results after transconjunctival resuturing of the scleral flap in hypotony following trabeculectomy. Am. J. Ophthalmol..

[45] Tunç Y., Tetikoglu M., Kara N., Sagdık H.M., Özarpaci S., Elçioğlu M.N. (2015). Management of hypotony and flat anterior chamber associated with glaucoma filtration surgery. Int. J. Ophthalmol..

[46] Cashwell L.F., Martin C.A. (1999). Axial length decrease accompanying successful glaucoma filtration surgery. Ophthalmology.

[47] Camp A.S., Weinreb R.N. (2020). Hypotony Keratopathy Following Trabeculectomy. J. Glaucoma.

[48] Azuma K., Saito H., Takao M., Araie M. (2020). Frequency of hypotonic maculopathy observed by spectral domain optical coherence tomography in post glaucoma filtration surgery eyes. Am. J. Ophthalmol. Case Rep..

[49] Saeedi O., Pillar A., Jefferys J., Arora K., Friedman D., Quigley H. (2014). Change in choroidal thickness and axial length with change in intraocular pressure after trabeculectomy. Br. J. Ophthalmol..

